# Cardiovascular risk factors associated with acute myocardial infarction and stroke in the MADIABETES cohort

**DOI:** 10.1038/s41598-021-94121-8

**Published:** 2021-07-27

**Authors:** M. A. Salinero-Fort, F. J. San Andrés-Rebollo, J. Cárdenas-Valladolid, M. Méndez-Bailón, R. M. Chico-Moraleja, E. Carrillo de Santa Pau, I. Jiménez-Trujillo, I. Gómez-Campelo, C. de Burgos Lunar, J. M. de Miguel-Yanes, MADIABETES, J. C. Abanades-Herranz, A. M. Sobrado-de Vicente-Tutor, Mar Sanz-Pascual, M. Arnalte-Barrera, S. Pulido-Fernández, E. M. Donaire-Jiménez, C. Montero-Lizana, M. Domínguez-Paniagua, P. Serrano-Simarro, R. Echegoyen-de Nicolás, P. Gil-Díaz, I. Cerrada-Somolinos, R. Martín-Cano, A. Cava-Rosado, T. Mesonero-Grandes, E. Gómez-Navarro, A. Maestro-Martín, A. Muñoz-Cildoz, M. E. Calonge-García, M. Martín-Bun, P. Carreño-Freire, J. Fernández-García, A. Morán-Escudero, J. Martínez-Irazusta, E. Calvo-García, A. M. Alayeto-Sánchez, C. Reyes-Madridejos, M. J. Bedoya-Frutos, B. López-Sabater, J. Innerarity-Martínez, A. Rosillo-González, A. I. Menéndez-Fernández, F. Mata-Benjumea, P. Vich-Pérez, C. Martín-Madrazo, M. J. Gomara-Martínez, C. Bello-González, A. Pinilla-Carrasco, M. Camarero-Shelly, A. Cano-Espin, J. Castro Martin, B. de Llama-Arauz, A. de Miguel-Ballano, M. A. García-Alonso, J. N. García-Pascual, M. I. González-García, C. López-Rodríguez, M. Miguel-Garzón, M. C. Montero-García, S. Muñoz-Quiros-Aliaga, S. Núñez-Palomo, O. Olmos-Carrasco, N. Pertierra-Galindo, G. Reviriego-Jaén, P. Rius-Fortea, G. Rodríguez-Castro, J. M. San Vicente-Rodríguez, M. E. Serrano-Serrano, M. M. Zamora-Gómez, M. P. Zazo-Lázaro

**Affiliations:** 1 Fundación de Investigación e Innovación Biosanitaria de Atención Primaria, Madrid, Spain; 2 Instituto de Investigación Sanitaria del Hospital Universitario la Paz (IdIPAZ), Madrid, Spain; 3 Red de Investigación en Servicios de Salud en Enfermedades Crónicas (REDISSEC), Madrid, Spain; 4 Subdirección General de Investigación Sanitaria y Documentación, Consejería de Sanidad, Madrid, Spain; 5 Centro de Salud Las Calesas, Gerencia Asistencial de Atención Primaria, Calle de Las Calesas, 12, 28026 Madrid, Spain; 6 Sistemas de Información, Gerencia Asistencial de Atención Primaria Madrid, Madrid, Spain; 7 Universidad Alfonso x El Sabio, Madrid, Spain; 8 Departamento de Medicina Interna, Hospital Universitario Clínico San Carlos, Facultad de Medicina, Universidad Complutense de Madrid (UCM), Instituto de Investigación Sanitaria Hospital Clínico de San Carlos (IdISSC), Madrid, Spain; 9 Hospital Central de la Defensa, Madrid, Spain; 10 Computational Biology Group, Precision Nutrition and Cancer Program, IMDEA Food Institute, Madrid, Spain; 11 Unidad Docente y de Investigación en Medicina Preventiva y Salud Pública, Facultad de Ciencias de La Salud, Universidad Rey Juan Carlos, Alcorcón, Madrid, Spain; 12 Departamento de Medicina Preventiva del Hospital Clínico de San Carlos, Instituto de Investigación Sanitaria Hospital Clínico de San Carlos (IdISSC), Madrid, Spain; 13 Departamento de Medicina Interna, Hospital General Universitario Gregorio Marañón. Facultad de Medicina, Universidad Complutense de Madrid (UCM), Instituto de Investigación Sanitaria Gregorio Marañón (IiSGM), Madrid, Spain

**Keywords:** Diabetes, Endocrinology, Cardiology, Myocardial infarction

## Abstract

We aimed to develop two models to estimate first AMI and stroke/TIA, respectively, in type 2 diabetes mellitus patients, by applying backward elimination to the following variables: age, sex, duration of diabetes, smoking, BMI, and use of antihyperglycemic drugs, statins, and aspirin. As time-varying covariates, we analyzed blood pressure, albuminuria, lipid profile, HbA1c, retinopathy, neuropathy, and atrial fibrillation (only in stroke/TIA model). Both models were stratified by antihypertensive drugs. We evaluated 2980 patients (52.8% women; 67.3 ± 11.2 years) with 24,159 person-years of follow-up. We recorded 114 cases of AMI and 185 cases of stroke/TIA. The factors that were independently associated with first AMI were age (≥ 75 years vs. < 75 years) (p = 0.019), higher HbA1c (> 64 mmol/mol vs. < 53 mmol/mol) (p = 0.003), HDL-cholesterol (0.90–1.81 mmol/L vs. < 0.90 mmol/L) (p = 0.002), and diastolic blood pressure (65–85 mmHg vs. < 65 mmHg) (p < 0.001). The factors that were independently associated with first stroke/TIA were age (≥ 75 years vs. < 60 years) (p < 0.001), atrial fibrillation (first year after the diagnosis vs. more than one year) (p = 0.001), glomerular filtration rate (per each 15 mL/min/1.73 m^2^ decrease) (p < 0.001), total cholesterol (3.88–6.46 mmol/L vs. < 3.88 mmol/L) (p < 0.001), triglycerides (per each increment of 1.13 mmol/L) (p = 0.031), albuminuria (p < 0.001), neuropathy (p = 0.01), and retinopathy (p = 0.023).

## Introduction

Epidemiological studies have long sought to identify the clinical variables that contribute to incident cardiovascular events^1^. Results from these reports have enabled the construction of tools to estimate individual cardiovascular risk based on clinical data^2^. However, a significant number of older large-scale longitudinal studies have followed up cohorts that are characteristic of the general population, whereas relatively few have focused specifically on people with diabetes^3,4^. Similarly, cardiovascular risk factors have been extensively characterized in the population as a whole. However, fewer data have been reported on the specific effects of traditional cardiovascular risk factors in people with type-2 diabetes mellitus (T2DM) in a large representative outpatient cohort from southern Europe. Such data could add to global knowledge.

The contribution of cardiovascular risk factors to the onset of clinical cardiovascular events and optimal targets when treating these factors might differ between the general and the T2DM populations^5,6^. Consequently, specific risk prediction tools for cardiovascular disease in people with T2DM have been developed^7,8^.

Another disadvantage of traditional epidemiological studies is that most cohorts are from the U.S. and western Europe, with the result that cardiovascular risk estimation tools seem to perform differently depending on the ethnic, geographical, and social characteristics of the population in which they are used^9,10^. In recent years, tools to assess the risk of cardiovascular events have been developed in Mediterranean countries, albeit in patients newly diagnosed with type 2 diabetes mellitus^11^.

Clinical risk factors associated with acute myocardial infarction and stroke may not completely overlap in the T2DM population. In a recent report from Sweden^12^, the most strongly associated factors for acute myocardial infarction and stroke were glycated hemoglobin level, systolic blood pressure, and physical activity. Regarding lipids, LDL cholesterol had a weaker effect on stroke than on myocardial infarction. For example, for an LDL cholesterol value close to 4 mmol/L, the risk of myocardial infarction increased by 50% (HR 1.50), while the risk for stroke barely reached 10%^12^, with 2.5 mmol/L as the reference category for these analyses.

Here, we aimed to identify clinical variables that were significantly associated with a first acute myocardial infarction and stroke or transient ischemic attack (TIA) in the T2DM cohort of the Madrid Diabetes Study (MADIABETES) in Spain. The patients analyzed were representative of a Mediterranean population.

## Results

In the MADIABETES cohort, we gathered data on 2980 people with T2DM who had not previously experienced a cardiovascular event (24,159 person-years of follow-up). Of these, 1408 patients were men (47.2%) and 1572 were women (52.8%). Mean age was 67.3 ± 11.2 years, with a mean duration of T2DM of 11.8 ± 9.8 years (Table 1). Average chronic glycemic control was good (mean HbA1c = 53.3 ± 11.9 mmol/mol, with one in every six patients already being treated with insulin and one third who had already had retinopathy, neuropathy or nephropathy. Three quarters of the patients had hypertension, and cardiovascular risk, as measured by the adjusted REGICOR risk function, was low (Table 1).

**Table 1:** Baseline characteristics of the MADIABETES cohort (total population, and according to incident first acute myocardial infarction and incident first stroke/transient ischemic attack).

	95% CI Total (n = 2980)		AMI (n = 114)	No AMI (n = 2866)	*p* value	Stroke/TIA (n = 185)	No Stroke/TIA (n = 2795)	*p* value
**Sociodemographic variables**								
Female sex (%)	52.8	50.9–54.6	45.6	53.0	0.120	61.1	52.2	0.019
Age (years), mean (SD)	67.3 (11.2)	66.9–67.7	68.5 (11.6)	67.2 (11.1)	0.220	72.6 (9.9)	66.9 (11.1)	< 0.001
Duration of diabetes (years), mean (SD)	11.8 (9.8)	11.4–12.1	12.3 (9.2)	11.7 (9.8)	0.515	14.1 (11.2)	11.6 (9.6)	0.001
Current smoker (%)	19.6	18.2–21.1	19.4	19.6	0.989	13.9	20.0	0.004
**Medication profile** (%)								
Lifestyle changes only	22.3	20.9–23.9	18.4	22.5	0.414	18.9	22.6	0.357
Oral antihyperglycemic drugs	61.1	59.4–62.9	61.4	61.1		61.6	61.1	
Insulin ± other antihyperglycemic drugs	16.5	15.2–17.9	20.2	16.4		19.5	16.4	
Antihypertensive agents	76.2	74.7–77.7	86.8	75.8	0.007	83.1	75.7	0.029
Aspirin	35.7	34.0–37.5	41.2	35.5	0.209	38.4	35.6	0.446
Statins	64.4	62.7–66.1	65.8	64.4	0.757	67.8	64.3	0.342
**History of** (%)								
Nephropathy^A^	27.1	25.5–28.7	35.1	26.7	0.049	43.8	25.9	< 0.001
Neuropathy	5.5	4.7–6.3	7.9	5.4	0.246	8.6	4.1	0.003
Retinopathy	6.1	5.3–7.0	7.9	6.0	0.416	9.2	5.9	0.071
Prior complications^B^	33.9	32.2–35.6	39.5	33.6	0.196	55.5	32.5	< 0.001
Hypertension	74.5	72.8–76.1	82.1	74.2	0.070	84.9	73.9	0.002
Atrial fibrillation	9.3	8.3–10.4	13.2	6	0.002	12.7	5.9	0.001
**Risk of coronary events**^**C**^								
10-year risk, mean (SD)	5.4 (2.7)	5.3–5.5	5.8 (3.1)	5.4 (2.7)	0.119	5.6 (2.7)	5.4 (2.8)	0.441
**Anthropometric variables**								
BMI (kg/m^2^), mean (SD)	30.4 (5.1)	30.2–30.6	30.5 (5.3)	30.4 (5.1)	0.917	29.9 (5.1)	30.5 (5.1)	0.188
SBP (mmHg), mean (SD)	133.3 (11.8)	132.8–133.7	135.9 (13.7)	133.2 (11.7)	0.015	134.1 (11.7)	133.2 (11.8)	0.302
DBP (mmHg), mean (SD)	76.6 (6.9)	76.4–76.9	76.1 (7.4)	76.7 (6.9)	0.371	76.2 (6.7)	76.7 (6.9)	0.389
**Laboratory variables**								
GFR (mL/min/1.73 m^2^), mean (SD)	73.8 (16.9)	73.2–74.4	72.4 (18.9)	73.8 (16.9)	0.385	67.2 (17.1)	74.3 (16.8)	< 0.001
HbA1c (%), mean (SD)	7.02 (1.1)	6.9–7.07	7.33 (1.4)	7.01 (1.08)	0.003	7.2 (1.2)	7.0 (1.1)	0.017
HbA1c (mmol/mol), mean (SD)	53.3 (11.9)	52.9–53.8	56.6 (15.1)	53.2(11.8)		55.4 (13)	53.2 (11.9)	
**Dyslipidemia**								
Total cholesterol (mmol/L), mean (SD)	4.95 (0.82)	4.92–4.98	4.99 (1.01)	4.95 (0.82)	0.622	190.5 (4.93)	191.7 (4.96)	0.616
LDL-C (mmol/L), mean (SD)	2.90 (0.66)	2.87–2.92	2.89 (0.73)	112 (2.89)	0.902	2.84 (0.72)	2.90 (0.66)	0.270
HDL-C (mmol/L), mean (SD)	1.30 (0.32)	1.29–1.31	1.25 (0.33)	1.30 (0.32)	0.092	1.31 (0.32)	1.30 (0.33)	0.733
Triglycerides (mmol/L), median (IQR)	1.39 (0.77)	1.36–1.41	1.32 (1.12)	1.39 (0.77)	0.700	1.38 (0.72)	1.39 (0.78)	0.755
Microalbuminuria (> 30 mg/dl) (%)	16.6	14.9–18.4	28.2	16.1	0.007	31.6	15.5	< 0.001
Achievement of ABC^D^ (%)	6.1	5.3–7.0	7.9	6.0	0.411	6.0	6.1	0.979

*AMI* Acute myocardial infarction, *TIA* Transient ischemic attack, *SD* Standard deviation, *BMI* Body mass index, *SBP* Systolic blood pressure, *DBP* Diastolic blood pressure, *GFR* Glomerular filtration rate, *HbA1c* Glycated hemoglobin, *LDL-C* Low-density lipoprotein cholesterol, *HDL-C* High-density lipoprotein cholesterol, *IQR* Interquartile range.

^A^Nephropathy was defined as GFR lower than 60 mL/min/1.73 m^2^ or albuminuria (> 30 mg/dL).

^B^Nephropathy and/or Neuropathy and/or Retinopathy.

^C^Risk of developing coronary events as defined by the adjusted REGICOR function^64^.

^D^Achievement of ABC: HbA1c < 7% + LDL-cholesterol < 2.6 mmol/L + Blood pressure < 130/80 mmHg.

### Incident first acute myocardial infarction

During follow-up, we recorded 114 cases of incident first acute myocardial infarction (62 cases in men and 52 cases in women). The crude and age-standardized incidence rates were 44.03 and 44.39 per 1,000 for males, respectively, and 33.08 and 31.38 per 1,000 for females, respectively (Supplementary Tables S1 and S2).

The incidence density rate of a first acute myocardial infarction was 4.72 cases (95% CI 3.89–5.67) per 1,000 person-years (5.52 [95% CI 4.23–7.08] in men and 4.02 [95% CI 3.00–5.27] in women).

**Figure 1:**
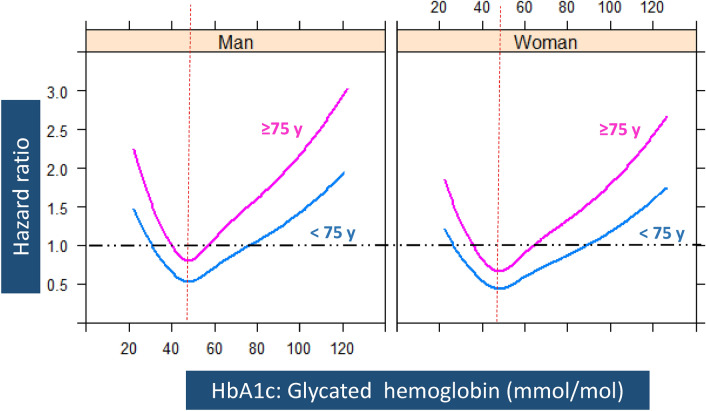
Representation of relationship between HbA1c* values and the Hazard Ratio of Myocardial Infarction, according to sex and age group. *Adjusted for HDL-cholesterol, DBP and albuminuria (Table 2). Dashed red lines indicate the hemoglobin value associated to a lower risk of AMI. Pink lines represent the adjusted effect of HbA1c on the risk of AMI in patients aged 75 years or older. Blue lines represent the adjusted effect of HbA1c on the risk of AMI in patients under 75 years of age.

In the bivariate analyses, people who had an incident first acute myocardial infarction showed higher values of systolic blood pressure despite being more often treated with antihypertensive drugs, higher HbA1c values and more frequently having atrial fibrillation, microalbuminuria, and prevalent nephropathy (all nominal p values < 0.05) (Table 1). We found the influence of sex on the association between HbA1c levels and incident first acute myocardial infarction to be heterogeneous: the risk for a man younger than 75 years was similar to that of a woman older than 75 years (Fig. 1).

### Incident first stroke/TIA

A total of 185 cases of incident first stroke or TIA were collected during follow-up (72 cases in men and 113 cases in women). The incidence density rate of a first stroke or TIA was 7.66 cases (95% CI 6.60–8.85) per 1000 person-years (6.42 [95% CI 5.02–8.08] in men, and 8.74 [95% CI 7.20–10.51] in women).

The crude cumulative and age-standardized stroke/TIA incidence was higher in females (71.88 and 67.06 per 1000, respectively) than in males (51.14 and 57.18 per 1000, respectively) (Supplementary Tables S3–S4).

In the bivariate analyses, people who had an incident first stroke or TIA were older and more frequently women and had a longer duration of diabetes and worse chronic glycemic control. Hypertension, atrial fibrillation, less active smoking, microalbuminuria, nephropathy, a lower glomerular filtration rate, and neuropathy were more prevalent among the people who had a first stroke or TIA during follow-up (all nominal p values < 0.05) (Table 1).

### Multivariable analysis of first acute myocardial infarction

In the multivariable model constructed for a first incident of acute myocardial infarction, the significantly highest hazard ratios were found for albuminuria > 300 mg/dL vs. < 30 mg/dL (HR 2.61; 95% CI, 1.04–6.52; p = 0.040), followed by HbA1c > 64 mmol/mol vs. < 53 mmol/mol (HR 1.95; 95% CI 1.24–3.04; p = 0.003), and age ≥ 75 years vs. < 75 years (HR 1.62; 95% CI 1.08–2.42; p = 0.019). On the other hand, the significantly strongest protective factors for acute myocardial infarction were HDL-cholesterol and DBP: HDL-cholesterol values between 0.90 and 1.81 mmol/L (HR 0.50; 95% CI 0.32–0.78; p = 0.002) compared with the reference category of HDL-cholesterol < 0.90 mmol/L; and DBP values between 65 and 80 mmHg (HR 0.41; 95% CI 0.26–0.63; p < 0.001) compared with values below 65 mmHg (Table 2). Supplementary Fig. S2 shows that within an approximate range of 65–85 mmHg, the risk is less than 1 (protective effect), while for values lower or higher than that interval, the risk is higher than 1.

**Table 2:** Multivariable analysis of factors associated with incident first acute myocardial infarction in the MADIABETES cohort.

Initial model					Final model^a^				
Risk factors	Hazard ratio	95% confidence interval		*p* value	Risk factors^b^	Hazard ratio	95% confidence interval		*p* value
Female sex	0.72	0.48	1.08	0.112	Female sex	0.74	0.50	1.09	0.122
Age (per each 1-year increment)	1.02	1.01	1.04	0.044	Age ≥ 75 years old (reference < 75 years)	1.62	1.08	2.42	0.019
HbA1c (per each 11-mmol/mol increment)	1.09	0.96	1.25	0.075	**HbA1c (reference < 53 mmol/mol)**				0.002
					53–64 mmol/mol	0.80	0.48	1.32	0.382
					> 64 mmol/mol	1.95	1.24	3.04	0.003
HDL-cholesterol (per each 0.03-mmol/L increment)	0.98	0.97	1.02	0.072	**HDL-cholesterol (reference < 0.90 mmol/L)**				0.010
					0.90–1.81 mmol/L	0.50	0.32	0.78	0.002
					> 1.81 mmol/L	0.58	0.25	1.34	0.203
DBP (per each 1-mmHg increment)	0.97	0.95	0.99	0.045	**DBP (reference < 65 mmHg)**				0.000
					65–85 mmHg	0.41	0.26	0.63	0.000
					> 85 mmHg	0.60	0.30	1.21	0.155
Albuminuria (per each 1-mg/dL increment)	1.01	0.98	1.04	0.082	**Albuminuria (reference < 30 mg/dL)**				0.114
					30–300 mg/dL	1.18	0.67	2.07	0.560
					> 300 mg/dL	2.61	1.04	6.52	0.040
BMI (per each 1-kg/m^2^ increment)	1.01	0.97	1.05	0.772					
Diabetes duration (per each 1-year increment)	1.00	0.98	1.02	0.936					
Current smoker (yes vs. no)	1.11	0.64	1.90	0.718					
Statins (yes vs. no)	0.95	0.64	1.42	0.797					
**Diabetes treatment (reference lifestyle changes)**				0.970					
Oral antihyperglycemic drugs	1.03	0.62	1.71	0.903					
Insulin ± other antihyperglycemic drugs	1.08	0.56	2.08	0.809					
Aspirin (yes vs. no)	1.08	0.73	1.60	0.689					
LDL-cholesterol (per each 0.03-mmol/L increment)	1.01	1.00	1.02	0.263					
Total cholesterol (per each 0.03-mmol/L increment)	1.00	0.99	1.01	0.487					
SBP (per each 1-mmHg increment)	1.01	0.99	1.02	0.315					
GFR (per each 1-mL/min/1.73 m^2^ increment)	1.00	0.99	1.01	0.496					
Retinopathy (yes vs. no)	1.27	0.74	2.17	0.384					
Neuropathy (yes vs. no)	1.25	0.66	2.35	0.497					
Triglycerides (per each 0.01-mmol/L increment)	1.00	0.99	1.00	0.225					
Antihypertensive treatment (yes vs. no)	1.92	1.07	3.44	0.028	Stratified variable^c^				

*BMI* Body mass index, *HbA1c* Glycated hemoglobin, *HDL-cholesterol* High-density lipoprotein cholesterol, *DBP* Diastolic blood pressure, *LDL-cholesterol* Low-density lipoprotein cholesterol, *SBP* Systolic blood pressure, *GFR* Glomerular filtration rate.

^a^Backward elimination (Supplementary Fig. S1).

^b^HDL-cholesterol, Albuminuria, DBP, and HbA1c were kept as continuous variables for descriptive analyses and the initial Cox regression model. Subsequently, the variables were categorized, given their non-linear effect on outcomes (Supplementary Figs. S2 and S3). Age was categorized according to clinical relevance.

^c^The inclusion as an independent variable produced inconsistent results given that the vast majority of the cardiovascular events were concentrated in the category of patients taking antihypertensive drugs. We repeated the analysis by stratifying by this variable that allows taking it into account in the adjustment and solving this problem.

### Multivariable analysis of first stroke or TIA

In the multivariable model constructed for a first incident stroke or TIA, the variables that were independently associated with onset of a first stroke or TIA were as follows: older age (HR 3.32; 95% CI 1.97–5.58; p < 0.001) for patients > 75 years vs. < 60 years as the reference category; atrial fibrillation, with a higher risk during the first year after the diagnosis (HR 2.64; 95% CI 1.45–4.82; p = 0.001); higher triglyceride levels (HR 1.20; 95% CI 1.02–1.41; p = 0.031) per each 1.13 mmol/L increase; albuminuria (HR 1.94; 95% CI 1.35–2.78; p < 0.001; glomerular filtration rate (HR 2.20; 95% CI 1.55–3.14; p < 0.001) per each 15 mL/min/1.73 m^2^ decrease; neuropathy (HR 1.73; 95% CI 1.14–2.64; p = 0.01), and retinopathy (HR 1.55; 95% CI 1.06–2.26; p = 0.023). However, total cholesterol levels between 3.88 and 6.46 mmol/L vs. total cholesterol levels < 3.88 mmol/L as the reference category showed an inverse and significant association with stroke or TIA (Table 3).

**Table 3:** Multivariable analysis of factors associated with incident first stroke/transient ischemic in the MADIABETES cohort.

Initial model					Final model^a^				
Risk factors	Hazard ratio	95% confidence interval		*p* value	Risk factors^b^	Hazard ratio	95% confidence interval		*p* value
Female sex vs. male sex	1.10	0.78	1.53	0.589	Female sex vs. male sex	1.21	0.88	1.66	0.248
Age (per each 1-year increment)	1.04	1.02	1.06	0.000	**Age (reference < 60 years old)**				< 0.001
					60–64 years old	1.79	0.96	3.33	0.068
					65–75 years old	2.10	1.25	3.53	0.005
					> 75 years old	3.32	1.97	5.58	< 0.001
BMI (per each 1-kg/m^2^ increment)	0.98	0.96	1.02	0.089	**Body mass index (reference < 25 kg/m**^**2**^)				0.085
					25–29.9 kg/m^2^	0.68	0.45	1.04	0.078
					30–35 kg/m^2^	0.55	0.35	0.87	0.011
					> 35 kg/m^2^	0.67	0.40	1.13	0.131
GFR (mL/min/1.73 m^2^)*	1.97	1.47	2.98	0.006	Glomerular filtration rate (GFR)*	2.20	1.55	3.14	< 0.001
Albuminuria (per each 1-mg/dL increment)	1.01	0.97	1.03	0.070	Albuminuria (> 30 mg/dL vs. ≤ 30 mg/dL)	1.94	1.35	2.78	< 0.001
Atrial fibrillation (yes vs. no)	1.81	1.21	2.72	0.004	**Atrial fibrillation**	1.71	1.14	2.55	0.010
					< 1-year onset	2.64	1.45	4.82	0.001
					1–4 years onset	1.59	1.04	2.42	0.031
					5–10 years onset	1.23	0.70	2.16	0.470
Neuropathy (yes vs. no)	1.84	1.21	2.81	0.005	Neuropathy (yes vs. no)	1.73	1.14	2.64	0.010
Retinopathy (yes vs. no)	1.61	1.09	2.39	0.016	Retinopathy (yes vs. no)	1.55	1.06	2.26	0.023
Triglycerides (per each 1.13-mmol/L increment)	1.30	1.10	1.50	0.013	Triglycerides (per each 1.13 mmol/L increment)	1.20	1.02	1.41	0.031
Total cholesterol (per each 0.03 mmol/L increment)	0.99	0.98	1.00	0.059	**Total cholesterol (reference < 3.88 mmol/L)**				0.001
					3.88–6.46 mmol/L	0.53	0.38	0.74	< 0.001
					> 6.46 mmol/L	0.41	0.14	1.19	0.102
HbA1c (per each 11-mmol/mol increment)	1.14	0.98	1.27	0.115	**HbA1c (reference < 53 mmol/mol**)				0.347
					53–64 mmol/mol	1.03	0.72	1.46	0.882
					> 64 mmol/mol	1.32	0.90	1.96	0.160
Current smoker (yes vs. no)	1.14	0.69	1.88	0.611					
Statins (yes vs. no)	1.16	0.83	1.61	0.389					
**Diabetes treatment (reference lifestyle changes)**				0.667					
Oral antihyperglycemic drugs	1.13	0.76	1.70	0.541					
Insulin ± other antihyperglycemic drugs	0.97	0.57	1.64	0.902					
Aspirin (yes vs. no)	0.96	0.70	1.31	0.788					
LDL-cholesterol (per each 0.03-mmol/L increment)	1.00	0.99	1.01	0.658					
HDL-cholesterol (per each 0.03-mmol/L increment)	1.00	0.99	1.02	0.539					
SBP (per each 1-mmHg increment)	1.00	0.99	1.01	0.566					
DBP (per each 1-mmHg increment)	1.01	0.99	1.03	0.522					
Diabetes duration (per each 1-year increment)	1.00	0.99	1.02	0.307					
Antihypertensive treatment (yes vs. no)	1.21	0.96	1.55	0.079	Stratified variable^c^				

*BMI* Body mass index, *HbA1c* Glycated hemoglobin, *HDL-cholesterol* High-density lipoprotein cholesterol, *LDL-cholesterol* Low-density lipoprotein, *SBP* Systolic blood pressure, *DBP* Diastolic blood pressure.

*GFR: Glomerular filtration rate per each 15 mL/min/1.73 m^2^ decrease.

^a^Backward elimination (Supplementary Fig. S1).

^b^BMI, albuminuria, and cholesterol were kept as continuous variables for the descriptive analyses and the initial model of Cox regression. Subsequently, the variables were categorized, given the non-linear effect de these variables on outcomes (Supplementary Figs. S2 and S3). Age and HbA1c were categorized according to clinical relevance.

^c^The inclusion as an independent variable produced inconsistent results given that the vast majority of the cardiovascular events were concentrated in the category of patients taking antihypertensive drugs. We repeated the analysis stratifying by this variable that allows taking it into account in the adjustment and solving this problem.

## Discussion

The present study reports data on the risk factors associated with the incidence of first acute myocardial infarction and stroke/TIA in a well-balanced Spanish type 2 diabetes cohort in terms of sex. Glycemic control was optimal (HbA1c = 53.3 mmol/mol) and cardiovascular risk low, as measured by the adjusted REGICOR risk function. Furthermore, as patients were from the primary care setting, they were not selected by degree of complexity, as usually happens with patients who require care in hospital or diabetes clinics. Moreover, our study differs from other European cohorts, including Mediterranean cohorts^13^, in the following items: lower baseline HbA1c level, lower insulin use, and less established microvascular complications such as retinopathy. We think that these differences with other European cohorts justify the present study, which may provide more comprehensive data on risk factors in patients with a low-risk profile.

We found incidence density rates of 4.72 cases/1000 person-years for first acute myocardial infarction and 7.66 cases/1000 person-years for first stroke or TIA in a primary care cohort of people with T2DM from a Mediterranean country who were free of cardiovascular events at baseline. Age independently predicted both first acute myocardial infarction and first stroke or TIA. Chronic poor glycemic control, low HDL-cholesterol, and low diastolic blood pressure were independently associated with first acute myocardial infarction alone, whilst atrial fibrillation, total cholesterol and triglyceride levels, albuminuria, lower glomerular filtration rate, neuropathy and retinopathy were exclusively associated with first stroke or TIA.

The cumulative incidence of myocardial infarction and stroke was lower in our study than in other, similar studies^14^, probably because our cohort comprises patients with a low risk of cardiovascular events, as measured using the REGICOR adjusted function (a calibration of the Framingham algorithm adapted for Spain)^15^.

Regarding incidence rates per 1000 person-years, it is difficult to compare our results with those of other investigations owing to the marked differences arising from the age of the participants in these studies. In the USA, Mozaffarian et al.^16^ reported rates for incident acute myocardial infarction ranging between 0.8 and 9.1 cases/1000 person-years among white men and between 0.2 and 7.8 cases/1000 person-years among white women according to age group. In the same study, rates for incident stroke/TIA ranged between 2.4 and 12.2 cases/1000 person-years among white men and between 2.4 and 9.9 cases/1000 person-years among white women according to age group. Holman et al.^17^ reported 16.8 cases of acute myocardial infarction/1000 person-years and 6.3 cases of stroke/1000 person-years in people with intensively treated T2DM. In the CAPAMIS study, a population-based cohort including 27,204 people older than 60 years in Tarragona (Spain)^18^, the incidence of acute myocardial infarction was 9.14 per 1000 person-years. The incidence was two-fold higher than in our cohort despite similar ages at baseline. A possible explanation for the differences between our study and CAPAMIS is that we excluded patients with previous cardiovascular events at baseline, with the result that the number of acute myocardial infarctions was low.

The role of lipid levels as predictors of coronary heart disease in the Mediterranean population has been highlighted by previous research. Orozco-Beltran et al.^19^ showed that low HDL-cholesterol (< 35 mg/dL) was the biochemical parameter with the strongest association with acute myocardial infarction in a Mediterranean population. Another study in the T2DM Spanish population showed that the ratio of non–HDL-cholesterol to HDL-cholesterol was a significant predictor of cardiovascular events, whilst no significant associations were observed for LDL-cholesterol and total cholesterol^11^. Likewise, the ERICE score, a new native cardiovascular score for the low-risk, elderly Mediterranean population of Spain, claimed that the contribution of serum total cholesterol to coronary heart disease was small^20^.

However, the effect of dyslipidemia on stroke is unclear, as has been shown in observational studies and a 1995 meta-analysis^21^ of 45 prospective cohorts. In fact, in 1994, D’Agostino et al.^22^ developed a prediction rule for the 10-year risk of stroke in primary prevention that did not include dyslipidemia. A further meta-analysis of observational studies on primary or secondary prevention of cardiovascular disease^23^ did not show an association between total serum cholesterol and fatal stroke. However, it did show a reduction in the cumulative incidence of stroke (total and non-fatal), probably owing to the lower risk of ischemic stroke, as reported elsewhere^24–26^.

The recent observational study by Rawshani et al.^12^ in patients with DM2 showed an association between LDL cholesterol and stroke. However, the relative importance for predicting stroke was weaker than for other variables, such as systolic blood pressure, atrial fibrillation, smoking, glycated hemoglobin, and duration of diabetes, and similar for diastolic blood pressure, body mass index, heart failure, and albuminuria.

On the other hand, the CARDS clinical trial in T2DM patients showed that lipids did not predict stroke^27^. However, the effect of statins on stroke in patients with diabetes has been observed in several clinical trials and confirmed in the Cholesterol Treatment Trialists' 2008 study^28^ on 18,686 people with diabetes in 14 randomized trials evaluating statins (OR = 0.79; 99% CI 0.67–0.93; p = 0.0002).

In our study, total cholesterol values between 3.88 and 6.46 mmol/L had a significant protective effect on stroke/TIA compared with levels lower than 3.88 mmol/L. This unexpected finding could mean that in primary prevention for patients with type 2 diabetes and low cardiovascular risk, intermediate total cholesterol values have incidence rates of ischemic stroke lower than < 3.88 mmol/L. In a similar sense, other studies^29^ in people with a low risk of vascular disease (< 5%) showed that, for each 1.0 mmol/L reduction in LDL cholesterol with statins, the RR (95% CI) for stroke was 0.74 (0.46–1.19), suggesting that the findings are not conclusive, given that the confidence interval includes a potential reduction in the incidence of stroke of 54% (appreciable benefit) or even an increase of 19% (harm).

The multinational INTERHEART Study emphasized that common risk factors underlie cardiovascular disease worldwide^30^, although variations in the attributable risk of each condition can be found depending on regional singularities.

We found that diastolic pressure lower than 65 mmHg was associated with a first acute myocardial infarction. This association has been described in previous studies ^31^ and is thought to be related to the fact that diastolic blood pressure below a critical level cannot ensure coronary flow during diastole^32^. Moreover, a similar J-shaped distribution was observed for values between 65–85 mmHg and > 85 mmHg. This distribution of DBP has previously been described for ischemic heart disease^33^. In Spain^34^, a reduction in the relative risk of cardiovascular disease has been described for values between 76 and 83 mmHg (RR = 0.75). The reduction is less intense for values ≥ 75 mmHg (RR = 0.88) with respect to DBP ≥ 84 mmHg. Our findings had a similar distribution, given that values < 65 mmHg were associated with a greater incidence of AMI than values between 65 and 85 mmHg (HR 0.41; 95% CI 0.26 to 0.63; p < 0.001) and values higher than 85 mmHg (HR 0.60; 95% CI 0.30–1.21; p = 0.155).

We also observed an association between HbA1c and incident acute myocardial infarction. The role of long-term close glycemic control to prevent macrovascular disease has been questioned by some reports^6^, even though most studies identify HbA1c to be independently associated with incident acute myocardial infarction^35^ and despite previous research underscoring the cardiovascular benefits of more intensive lowering of HbA1c^36^. In our study, HbA1c levels of around 48 mmol/mol were associated with the lowest risk of incident first acute myocardial infarction. This HbA1c goal is supported by some authors^37,38^, whereas other guidelines or recommendations claim less stringent values^39,40^. Our opinion is that such a strict target can only be considered adequate in older adults treated with diet alone or with drugs that do not cause hypoglycemia. The American Diabetes Association recommends long-term intensive diabetes management in patients who can be expected to live long enough to reap the benefits and whose cognitive and physical function is good^41^. Furthermore, recent European guidelines^42^ point out that HbA1c targets should be individualized, with more stringent goals (6.0–6.5% [42–48 mmol/mol]) in younger patients with a short duration of diabetes mellitus and no evidence of cardiovascular disease, if achieved without significant hypoglycemia.

Although diabetic neuropathy has been associated with myocardial ischemia^43^, in which endothelial dysfunction may be an underlying pathophysiological mechanism^44^, our findings did not replicate this finding. However, we did find neuropathy to be an independent predictor of incident first stroke or TIA, in accordance with other studies that had previously found an association with combined cardiovascular events (first acute myocardial infarction, cardiac revascularization, heart failure, stroke or TIA)^45^. Diabetic retinopathy has been associated with incident cardiovascular disease after adjusting for other microvascular and macrovascular complications^46^. To the best of our knowledge, no studies have associated retinopathy specifically with stroke or TIA, despite the reported association between diabetic retinopathy and acute myocardial infarction^47^.

The risk of stroke was highest during the first year after diagnosis of atrial fibrillation and decreased thereafter. This phenomenon has been found in other studies^48^ and could arise from the difficulty associated with achieving an optimal INR range during the first months after diagnosis.

Compared with other studies, we did not find any statistically significant association between active smoking and cardiovascular events^30,49^. This finding was unexpected, and we are unable to explain it. However, findings from the U.K. Clinical Practice Research Datalink^50^ have shown differences in the effects of smoking on stroke and myocardial infarction according to metformin use in type 2 diabetes mellitus patients. The hazard ratio for stroke or myocardial infarction was non-significant in current smokers receiving metformin (HR 1.08; 95% CI 0.81–1.45) compared with non-smokers not taking metformin. Therefore, concomitant treatment with metformin attenuates the higher cardiovascular and mortality risk observed in current smokers. In our study, more than 60% of patients received metformin, thus partially explaining the lack of a significant association with smoking.

We were not surprised to see that clinical factors did not overlap to predict acute myocardial infarction and stroke/TIA. As previously mentioned, Rawshani's study showed different risk patterns for stroke and myocardial infarction^12^. From a pathophysiology standpoint, it makes sense that different risk factors could be associated with each entity: in acute myocardial infarction, plaque rupture and in situ thrombosis are responsible for nearly all cases of transmural ischemia, thus conferring a heavier burden on atherothrombogenic factors; in stroke, factors other than plaque rupture are involved when the source is cardioembolic, in situ thrombosis, or lacunar infarct^51^.

In a classical epidemiological study of people with T2DM in Italy, researchers found that age, smoking, total/HDL-cholesterol, and HOMA-IR were the factors that best predicted incident cardiovascular disease^52^. Interesting studies have underscored the potential benefits of the Mediterranean diet and lifestyle for lowering incident cardiovascular events^53^. Cardiovascular risk factors may behave differently between populations, partly due to specific genetic background, but also due to specific environmental conditions. The interaction between factors might change the influence that each risk factor exerts on the eventual development of clinical cardiovascular disease.

Our study is subject to a series of limitations. The sample was not population-based, but rather drawn from outpatients seen in primary care centers of the Spanish Public Health System. The study was conducted in Madrid, which may not represent the entire Spanish population in terms of income, educational level, lifestyle, and health profile. The target population of this investigation was free of cardiovascular events at baseline; therefore, results might have been different if sought in secondary prevention. Residual confounding due to heterogeneous adherence to medical therapies could not be completely ruled out. A specific limitation of our study was the fact that we did not include socioeconomic status, educational level, physical activity, or alcohol consumption, which would at least have served as adjustment variables. Lastly, the loss follow-up rate was high. We had to take the last follow-up value as the data for analysis for patients who were lost to follow-up. These uncontrollable factors may have led to bias, and the external validity could be restricted to a certain extent.

However, the meticulous and prospective design of the study strengthens our findings. In addition, the fact that data are obtained from current medical practice in our setting provides us with an overall view of actual outcomes in patients with T2DM in our daily clinical practice.

## Conclusion

In our study of a Mediterranean T2DM population that was free of cardiovascular events at baseline, incidence density rates for first acute myocardial infarction and first stroke/transient ischemic attack were 4.72 cases and 7.66 cases/1000 person-years, respectively. Age was the only factor independently associated with both outcomes, since none of the remaining clinical factors were simultaneously associated with both outcomes. Additional research is needed to fully characterize predictors of cardiovascular disease in Mediterranean people with prevalent T2DM.

## Methods

MADIABETES is a large prospective dynamic cohort study whose characteristics have been described elsewhere^54^. The first recruitment drive took place in 2007 and enrolled 3443 T2DM outpatients; by the end of 2010, a second recruitment drive added a further 727 T2DM outpatients. The study population was recruited by simple random sampling from primary health care centers in the metropolitan area of Madrid (Spain): 56 patients in the first recruitment drive and 41 in the second. During the follow-up period (2008–2017), general practitioners collected data at the baseline visit (2007 and 2010, respectively) and annually thereafter under conditions of daily clinical practice. The baseline and prospective diagnoses were recorded in the case report form based on the information registered in electronic clinical records and/or hospital discharge reports.

The MADIABETES cohort is the Spanish type 2 diabetes mellitus cohort with the highest number of person-years of follow-up. It is also one of the few Spanish cohorts comprising primary care patients. Findings for the variables recorded—age, sex, time since diagnosis of diabetes, hypertension, dyslipidemia, and microvascular complications—are similar to those of Spanish hospital-based studies^55,56^ and the data reported by Bodicoat et al.^57^.

This cohort study was approved by the Institutional Review Board of Ramón y Cajal Hospital (Madrid) and conducted according to the principles of the Declaration of Helsinki. All patients gave their written informed consent to participate.

The database is not available to the public and its use is restricted to research groups affiliated with the MADIABETES consortium, IdIPAZ Health Research Institute, La Paz University Hospital, and IMDEA Food Institute, Madrid (Spain).

The inclusion criteria were age ≥ 30 years and a previous diagnosis of T2DM. We excluded homebound people and patients with type 1 diabetes mellitus.

Type 2 diabetes mellitus was defined based on a reported history of diabetes and/or according to the use of medications to treat diabetes and/or fasting plasma glucose > 125 mg/dL or two‐hour glucose > 200 mg/dL during an oral glucose tolerance test.

HbA1c was measured using the IFCC-calibrated method, as implemented by the central laboratory. We calculated body mass index (BMI) as weight/height^2^ (kg/m^2^). Blood pressure was measured twice using a checked, calibrated sphygmomanometer.

Neuropathy was defined as the presence of at least two of the following three criteria: abnormal sensory and/or motor signs, neuropathic symptoms, and absent or decreased tendon reflexes.

Retinopathy was based on the severity scale proposed by Wilkinson et al.^58^, as used previously by our group^54^. The scale consists of the presence of any of the following lesions: microaneurysm, intraretinal hemorrhage, venous beading, neovascularization, vitreous/preretinal hemorrhage, cotton wool spots, retinal thickening, and hard exudates.

Information on cigarette smoking was obtained by asking about current and lifetime smoking habits. Current or active smoking was defined as regular cigarette smoking (duration longer than six months) at the time of the examination. Former smoking was defined as a history of smoking for longer than six months and no current smoking. Never smoking was defined as no present or previous history of tobacco smoking.

Missing values were replaced by the last available value closest to the event incidence following the criterion of Altman et al.^59^.

### Outcomes

We developed separate models to explain two outcomes: (a) first event of fatal or non-fatal acute myocardial infarction; and (b) first event of fatal or non-fatal ischemic stroke or TIA.

Patients with a history of angina pectoris, myocardial infarction, stroke, TIA, and peripheral arterial disease (or amputation) registered in the electronic clinical records and/or hospital discharge reports were not included in the analysis cohort. For this analysis, only primary prevention patients were included.

Myocardial infarction was defined according to the Third Universal Definition of Myocardial Infarction^60^, as this was the definition at the time of the study and/or diagnosis documented in the clinical records.

Stroke was defined as rapid onset of clinical signs of focal or global loss of cerebral function that lasted for > 24 h and that could not be explained by other medical conditions and/or confirmed with brain computerized tomography (CT) scan or magnetic resonance imaging (MRI). Transient ischemic attack (TIA) was defined as the occurrence of neurological symptoms or signs that lasted < 24 h due to an episode of neurological dysfunction caused by focal brain, spinal cord, or retinal ischemia, without acute infarction^61^.

Angina pectoris was defined as substernal pain or discomfort (with or without radiation to other areas) precipitated by exercise and relieved by rest or sublingual nitroglycerine.

Peripheral arterial disease was defined as the presence of claudication, a history of peripheral artery surgery, and/or an ankle-brachial index less than 0.90 in either leg.

The vital status of each patient (dead or alive) was ascertained on December 31st, 2017, with data from the mortality records of the Spanish National Institute of Statistics (Instituto Nacional de Estadística, http://www.ine.es). Therefore, data about vital status and date and cause of death were available for all patients, with no loss of data during follow-up. The underlying cause of death stated on the death certificates was coded according to the International Statistical Classification of Diseases, Tenth Revision^62^.

### Statistical analysis

We report descriptive data as mean and standard deviation or median and interquartile range. We compared continuous variables between two groups using the *t* test for normally distributed data and the Mann–Whitney test for non-normally distributed data. Categorical variables were compared using the chi-squared test.

We calculated the cumulative incidence of first acute myocardial infarction and first stroke/TIA by taking the number of new cases as the numerator and the total initial population at risk as the denominator. We determined the number of patient-years at risk of developing first acute myocardial infarction or first stroke/TIA between the baseline appointment date and the date of death, the date of the event of a first acute myocardial infarction or first stroke/TIA, the date of loss to follow-up, or the end of the study period. We estimated incidence density as the number of new cases divided by patient-years at risk. The median follow-up was 9.9 years. We found that 11.4% of patients died and 25.8% were lost to follow-up due to loss of contact (median follow-up, 6.9 years).

The sex- and age-standardized incidence rates were calculated by the direct method based on the age distribution of the entire study population as the reference (Supplementary Tables S1–S4).

The objective of our study was not to build a predictive model. We aimed to explain the risk factors most frequently associated with the incidence of AMI and stroke/TIA in patients with type 2 diabetes using clinical, statistical, and pragmatic criteria. We used a multivariable extension of Cox proportional hazard analysis to estimate the adjusted hazard ratios (HRs) and corresponding 95% confidence intervals (CIs).

Initially, we constructed a multivariable model by applying backward elimination (PIN = 0.05, POUT = 0.2, based on the log-likelihood ratio test) (Supplementary Fig. S1) to a set of candidate predictors chosen as potential risk and confounding factors according to previous studies^63^. The data tested in the two models were as follows: age, sex, duration of diabetes, smoking, BMI, use of antihyperglycemic drugs (insulin, oral antidiabetic agents), statins, and aspirin. The time-varying covariates were systolic and diastolic blood pressure, albuminuria, lipid profile, HbA1c, retinopathy, neuropathy, and atrial fibrillation (only in stroke/TIA model). Both models were stratified by antihypertensive drugs.

The main interaction variables (age, sex) were tested using the chunk test, although the differences were not statistically significant. We checked the proportional hazards assumptions by examining the Schoenfeld residuals and the covariates ∗ time interaction terms and the assumption of a log-linear relationship between the predictors and the hazard function using the residual Martingale. The variables did not violate the assumption of proportional risks in either model, except for atrial fibrillation in the stroke/TIA model. For this variable, hazard ratios were calculated in different monitoring periods (< 1, 1–4, 5–10 years).

HbA1c, HDL-cholesterol, total cholesterol, diastolic blood pressure, albuminuria, and BMI violated the assumption of a log-linear relationship. We considered it more appropriate and practical to categorize the continuous variables that violated this assumption. In addition to clinical criteria, the visual analysis of these variables using the restricted cubic splines function guided us in establishing the most appropriate categories (Supplementary Figs. S2–S3).

A sensitivity analysis was performed after excluding stroke cases until three months after diagnosis of atrial fibrillation in order to study the real effect of atrial fibrillation on stroke.

We found no influential values (DFBETA index) or colinearity issues that significantly affected the results.

We evaluated the predictive accuracy of the multivariable Cox model using a bootstrapping method based on the Harrell C index, which is equivalent to the area under the receiver operating characteristic curve for binary dependent variables and replaces time-varying covariates by average values. Although the C index of the model for stroke/TIA (0.70) was somewhat greater than that of AIM (0.66), the discriminatory capacity of both models was weak. The analysis was performed using SPSS (version 21.0; IBM Corp, Armonk, NY, USA) and the “survival” and “rms” packages of R (GNU General Public License, version 3.5.1) (www.cran.r-project.org).

## Supplementary Information

Supplementary Information.

## References

[1] https://www.framinghamheartstudy.org/fhs-about/research-milestones. Accessed 9 Jan 2021.

[2] Anderson KM, Odell PM, Wilson PW, Kannel WB (1991). Cardiovascular disease risk profiles. Am. Heart J..

[3] The DCCT Research Group (1986). The Diabetes Control and Complications Trial (DCCT): Design and methodologic considerations for the feasibility phase. Diabetes.

[4] UK Prospective Diabetes Study (UKPDS) Group (1991). UK prospective diabetes study. VIII. Study design progress and performance. Diabetologia.

[5] The SPRINT Research Group (2015). A randomized trial of intensive versus standard blood-pressure control. N. Engl. J. Med..

[6] The ACCORD Study Group (2010). Effects of intensive blood-pressure control in type 2 diabetes mellitus. N. Engl. J. Med..

[7] Stevens RJ, Kothari V, Adler AI, Stratton IM, United Kingdom Prospective Diabetes Study (UKPDS) Group (2001). The UKPDS risk engine: a model for the risk of coronary heart disease in Type II diabetes (UKPDS 56). Clin. Sci..

[8] Kothari V, Stevens RJ, Adler AI, Stratton IM, Manley SE (2002). UKPDS 60: Risk of stroke in type 2 diabetes estimated by the UK Prospective Diabetes Study risk engine. Stroke.

[9] Tao L, Wilson EC, Griffin SJ, Simmons RK, ADDITION-Europe Study Team (2013). Performance of the UKPDS outcomes model for prediction of myocardial infarction and stroke in the ADDITION-Europe trial cohort. Value Health.

[10] Kengne AP, Patel A, Colagiuri S, Heller S, Hamet P (2010). The Framingham and UK Prospective Diabetes Study (UKPDS) risk equations do not reliably estimate the probability of cardiovascular events in a large ethnically diverse sample of patients with diabetes: the Action in Diabetes and Vascular Disease: Preterax and Diamicron-MR Controlled Evaluation (ADVANCE) Study. Diabetologia.

[11] Piniés JA, González-Carril F, Arteagoitia JM, Irigoien I, Altzibar JM, Rodriguez-Murua JL (2014). Development of a prediction model for fatal and non-fatal coronary heart disease and cardiovascular disease in patients with newly diagnosed type 2 diabetes mellitus: The Basque Country Prospective Complications and Mortality Study risk engine (BASCORE). Diabetologia.

[12] Rawshani A, Rawshani A, Franzén S, Sattar N, Eliasson B (2018). Risk factors, mortality, and cardiovascular outcomes in patients with type 2 diabetes. N. Engl. J. Med..

[13] Di Paola R, Marucci A, Fontana A, Menzaghi C, Salvemini L, Copetti M (2013). Role of obesity on all-cause mortality in whites with type 2 diabetes from Italy. Acta Diabetol..

[14] Novella B, Alonso M, Rodriguez-Salvanés F, Susi R, Reviriego B, Escalante L (2008). Incidencia a diez años de infarto de miocardio fatal y no fatal en la población anciana de Madrid [Ten-year incidence of fatal and non-fatal myocardial infarction in the elderly population of Madrid]. Rev. Esp. Cardiol..

[15] Marrugat J, Solanas P, D'Agostino R, Sullivan L, Ordovas J, Cordón F (2003). Estimación del riesgo coronario en España mediante la ecuación de Framingham calibrada [Coronary risk estimation in Spain using a calibrated Framingham function]. Rev. Esp. Cardiol..

[16] Mozaffarian D, Benjamin EJ, Go AS, Arnett DK, Blaha MJ (2015). Heart disease and stroke statistics-2015 update: A report from the American Heart Association. Circulation.

[17] Holman RR, Paul SK, Bethel MA, Matthews DR, Neil HA (2008). 10-year follow-up of intensive glucose control in type 2 diabetes. N. Engl. J. Med..

[18] Forcadell MJ, Vila-Córcoles A, de Diego C, Ochoa-Gondar O, Satué E (2018). Incidence and mortality of myocardial infarction among Catalonian older adults with and without underlying risk conditions: The CAPAMIS study. Eur. J. Prev. Cardiol..

[19] Orozco-Beltran D, Gil-Guillen VF, Redon J, Martin-Moreno JM, Pallares-Carratala V (2017). Lipid profile, cardiovascular disease and mortality in a Mediterranean high-risk population: The ESCARVAL-RISK study. PLoS ONE.

[20] Gabriel R, Brotons C, Tormo MJ, Segura A, Rigo F (2015). The ERICE-score: The new native cardiovascular score for the low-risk and aged Mediterranean population of Spain. Rev. Esp. Cardiol..

[21] Cholesterol, diastolic blood pressure, and stroke: 13,000 strokes in 450,000 people in 45 prospective cohorts: Prospective studies collaboration. *Lancet* **346**(8991–8992), 1647–1653 (1995). 8551820

[22] D'Agostino RB, Wolf PA, Belanger AJ, Kannel WB (1994). Stroke risk profile: Adjustment for antihypertensive medication: The Framinham Study. Stroke.

[23] Lewington S, Whitlock G, Clarke R, Sherliker P, Emberson J, Halsey J (2007). Blood cholesterol and vascular mortality by age, sex, and blood pressure: A meta-analysis of individual data from 61 prospective studies with 55,000 vascular deaths. Lancet.

[24] De Caterina R, Scarano M, Marfisi R, Lucisano G, Palma F, Tatasciore A (2010). Cholesterol-lowering interventions and stroke: Insights from a meta-analysis of randomized controlled trials. J. Am. Coll. Cardiol..

[25] Naci H, Brugts JJ, Fleurence R, Ades AE (2013). Comparative effects of statins on major cerebrovascular events: A multiple-treatments meta-analysis of placebo-controlled and active-comparator trials. QJM.

[26] Wang W, Zhang B (2014). Statins for the prevention of stroke: A meta-analysis of randomized controlled trials. PLoS ONE.

[27] Hitman GA, Colhoun H, Newman C, Szarek M, Betteridge DJ, Durrington PN (2007). Stroke prediction and stroke prevention with atorvastatin in the Collaborative Atorvastatin Diabetes Study (CARDS). Diabet. Med..

[28] Kearney PM, Blackwell L, Collins R, Keech A, Simes J, Peto R, Cholesterol Treatment Trialists' (CTT) Collaborators (2008). Efficacy of cholesterol-lowering therapy in 18,686 people with diabetes in 14 randomised trials of statins: A meta-analysis. Lancet.

[29] Mihaylova B, Emberson J, Blackwell L, Keech A, Simes J, Barnes EH, Cholesterol Treatment Trialists' (CTT) Collaborators (2012). The effects of lowering LDL cholesterol with statin therapy in people at low risk of vascular disease: Meta-analysis of individual data from 27 randomised trials. Lancet.

[30] Yusuf S, Hawken S, Ounpuu S, Dans T, Avezum A (2004). Effect of potentially modifiable risk factors associated with myocardial infarction in 52 countries (the INTERHEART study): Case-control study. Lancet.

[31] Zhao W, Katzmarzyk PT, Horswell R, Wang Y, Li W (2013). Aggressive blood pressure control increases coronary heart disease risk among diabetic patients. Diabetes Care.

[32] Messerli FH, Panjrath GS (2009). The J-curve between blood pressure and coronary artery disease or essential hypertension: Exactly how essential?. J. Am. Coll. Cardiol..

[33] Pastor-Barriuso R, Banegas JR, Damián J, Appel LJ, Guallar E (2003). Systolic blood pressure, diastolic blood pressure, and pulse pressure: An evaluation of their joint effect on mortality. Ann. Intern. Med..

[34] Baena-Díez JM, Bermúdez-Chillida N, García-Lareo M, Olivia Byram A, Vidal-Solsona M, Vilató-García M (2008). Papel de la presión de pulso, presión arterial sistólica y presión arterial diastólica en la predicción del riesgo cardiovascular. Estudio de cohortes [Role of pulse pressure, systolic blood pressure, and diastolic blood pressure in the prediction of cardiovascular risk. Cohort study]. Med Clin.

[35] Stratton IM, Cull CA, Adler AI, Matthews DR, Neil HA (2006). Additive effects of glycaemia and blood pressure exposure on risk of complications in type 2 diabetes: A prospective observational study (UKPDS 75). Diabetologia.

[36] Gaede P, Lund-Andersen H, Parving H-H, Pedersen O (2008). Effect of a multifactorial intervention on mortality in type 2 diabetes. N. Engl. J. Med..

[37] National Institute for Health and Care Excellence (2015; last updated 16 December 2020) Type 2 diabetes in adults: management (NICE guideline 28). https://www.nice.org.uk/guidance/ng28/chapter/Recommendations#hba1c-measurement-and-targets. Accessed 23 Dec 2020.

[38] Handelsman Y, Bloomgarden ZT, Grunberger G, Umpierrez G, Zimmerman RS (2015). American Association of Clinical Endocrinologists and American College of Endocrinology: Clinical practice guidelines for developing a diabetes mellitus comprehensive care plan: 2015. Endocr. Pract..

[39] Qaseem A, Wilt TJ, Kansagara D, Horwitch C, Barry MJ (2018). Hemoglobin A1c targets for glycemic control with pharmacologic therapy for nonpregnant adults with type 2 diabetes mellitus: A guidance statement update from the American College of Physicians. Ann. Intern. Med..

[40] U.S. Department of Veteran Affairs, U.S. Department of Defense. VA/DoD clinical practice guidelines: Management of diabetes mellitus in primary care. Updated April 18, 2017. https://www.healthquality.va.gov/guidelines/CD/diabetes/. Accessed 9 Jan 2021.

[41] American Diabetes Association (2021). Glycemic targets: Standards of medical care in diabetes-2021. Diabetes Care.

[42] Cosentino F, Grant PJ, Aboyans V, Bailey CJ, Ceriello A, Delgado V, ESC Scientific Document Group (2020). 2019 ESC Guidelines on diabetes, pre-diabetes, and cardiovascular diseases developed in collaboration with the EASD. Eur. Heart J..

[43] Baltzis D, Roustit M, Grammatikopoulou MG, Katsaboukas D, Athanasiou V (2016). Diabetic peripheral neuropathy as a predictor of asymptomatic myocardial ischemia in type 2 diabetes mellitus: A cross-sectional study. Adv. Ther..

[44] Roustit M, Loader J, Deusenbery C, Baltzis D, Veves A (2016). Endothelial dysfunction as a link between cardiovascular risk factors and peripheral neuropathy in diabetes. J. Clin. Endocrinol. Metab..

[45] Brownrigg JR, de Lusignan S, McGovern A, Hughes C, Thompson MM (2014). Peripheral neuropathy and the risk of cardiovascular events in type 2 diabetes mellitus. Heart.

[46] Gimeno-Orna JA, Faure-Nogueras E, Castro-Alonso FJ, Boned-Juliani B (2009). Ability of retinopathy to predict cardiovascular disease in patients with type 2 diabetes mellitus. Am. J. Cardiol..

[47] Miettinen H, Haffner SM, Lehto S, Rönnemaa T, Pyörälà K (1996). Retinopathy predicts coronary heart disease events in NIDDM patients. Diabetes Care.

[48] Frost L, Engholm G, Johnsen S, Møller H, Husted S (2000). Incident stroke after discharge from the hospital with a diagnosis of atrial fibrillation. Am. J. Med..

[49] O'Donnell MJ, Chin SL, Rangarajan S, Xavier D, Liu L, Zhang H (2016). Global and regional effects of potentially modifiable risk factors associated with acute stroke in 32 countries (INTERSTROKE): a case-control study. Lancet.

[50] Paul SK, Klein K, Majeed A, Khunti K (2016). Association of smoking and concomitant metformin use with cardiovascular events and mortality in people newly diagnosed with type 2 diabetes. J. Diabetes.

[51] Widimsky P, Coram R, Abou-Chebl A (2014). Reperfusion therapy of acute ischaemic stroke and acute myocardial infarction: similarities and differences. Eur. Heart J..

[52] Bonora E, Formentini G, Calcaterra F, Lombardi S, Marini F (2002). HOMA-estimated insulin resistance is an independent predictor of cardiovascular disease in type 2 diabetic subjects: prospective data from the Verona Diabetes Complications Study. Diabetes Care.

[53] Estruch R, Ros E, Salas-Salvadó J, Covas MI, Corella D (2018). Primary prevention of cardiovascular disease with a Mediterranean diet supplemented with extra-virgin olive oil or nuts. N. Engl. J. Med..

[54] Salinero-Fort MA, San Andrés-Rebollo FJ, de Burgos-Lunar C, Arrieta-Blanco FJ, Gómez-Campelo P (2013). Four-year incidence of diabetic retinopathy in a Spanish cohort: The MADIABETES study. PLoS ONE.

[55] Simó R, Bañeras J, Hernández C, Rodríguez-Palomares J, Valente F, Gutierrez L (2019). Diabetic retinopathy as an independent predictor of subclinical cardiovascular disease: Baseline results of the PRECISED study. BMJ Open Diabetes Res Care.

[56] Alvarez-Guisasola F, Cebrián-Cuenca AM, Cos X, Ruiz-Quintero M, Millaruelo JM, Cahn A (2018). Calculating individualized glycaemic targets using an algorithm based on expert worldwide diabetologists: Implications in real-life clinical practice. Diabetes Metab. Res. Rev..

[57] Bodicoat DH, Mundet X, Davies MJ, Khunti K, Roura P, Franch J (2015). The impact of a programme to improve quality of care for people with type 2 diabetes on hard to reach groups: The GEDAPS study. Prim. Care Diabetes.

[58] Wilkinson CP, Ferris FL, Klein RE, Lee PP, Agardh CD, Davis M, Global Diabetic Retinopathy Project Group (2003). Proposed international clinical diabetic retinopathy and diabetic macular edema disease severity scales. Ophthalmology.

[59] Altman DG, De Stavola BL (1994). Practical problems in fitting a proportional hazards model to data with updated measurements of the covariates. Stat. Med..

[60] Thygesen K, Alpert JS, Jaffe AS, Simoons ML, Chaitman BR, White HD, Joint ESC/ACCF/AHA/WHF Task Force for the Universal Definition of Myocardial Infarction (2012). Third universal definition of myocardial infarction. Circulation.

[61] Easton JD, Saver JL, Albers GW, Alberts MJ, Chaturvedi S, Feldmann E (2009). The American Academy of Neurology affirms the value of this statement as an educational tool for neurologists. Stroke.

[62] World Health Organization. *ICD-10: International Statistical Classification of Diseases and Related Health Problems: Tenth Revision*, 2nd edn. (World Health Organization, 2004).

[63] Dawber TR, Kannel WB, Revotskie N, Stokes J, Kagan A (1959). Some factors associated with the development of coronary heart disease: Six years' follow-up experience in the Framingham study. Am. J. Public Health Nations Health.

[64] Marrugat J, Subirana I, Comín E (2007). Validity of an adaptation of the Framingham cardiovascular risk function: The VERIFICA study. Investig. J. Epidemiol. Community Health..

