# Author Correction: Cardiovascular risk factors associated with acute myocardial infarction and stroke in the MADIABETES cohort

**DOI:** 10.1038/s41598-022-10213-z

**Published:** 2022-04-08

**Authors:** M. A. Salinero-Fort, F. J. San Andrés-Rebollo, J. Cárdenas-Valladolid, M. Méndez-Bailón, R. M. Chico-Moraleja, E. Carrillo de Santa Pau, I. Jiménez-Trujillo, I. Gómez-Campelo, C. de Burgos Lunar, J. M. de Miguel-Yanes, MADIABETES, J. C. Abanades-Herranz, A. M. Sobrado-de Vicente-Tutor, Mar Sanz-Pascual, M. Arnalte-Barrera, S. Pulido-Fernández, E. M. Donaire-Jiménez, C. Montero-Lizana, M. Domínguez-Paniagua, P. Serrano-Simarro, R. Echegoyen-de Nicolás, P. Gil-Díaz, I. Cerrada-Somolinos, R. Martín-Cano, A. Cava-Rosado, T. Mesonero-Grandes, E. Gómez-Navarro, A. Maestro-Martín, A. Muñoz-Cildoz, M. E. Calonge-García, M. Martín-Bun, P. Carreño-Freire, J. Fernández-García, A. Morán-Escudero, J. Martínez-Irazusta, E. Calvo-García, A. M. Alayeto-Sánchez, C. Reyes-Madridejos, M. J. Bedoya-Frutos, B. López-Sabater, J. Innerarity-Martínez, A. Rosillo-González, A. I. Menéndez-Fernández, F. Mata-Benjumea, P. Vich-Pérez, C. Martín-Madrazo, M. J. Gomara-Martínez, C. Bello-González, A. Pinilla-Carrasco, M. Camarero-Shelly, A. Cano-Espin, J. Castro Martin, B. de Llama-Arauz, A. de Miguel-Ballano, M. A. García-Alonso, J. N. García-Pascual, M. I. González-García, C. López-Rodríguez, M. Miguel-Garzón, M. C. Montero-García, S. Muñoz-Quiros-Aliaga, S. Núñez-Palomo, O. Olmos-Carrasco, N. Pertierra-Galindo, G. Reviriego-Jaén, P. Rius-Fortea, G. Rodríguez-Castro, J. M. San Vicente-Rodríguez, M. E. Serrano-Serrano, M. M. Zamora-Gómez, M. P. Zazo-Lázaro

**Affiliations:** 1 Fundación de Investigación e Innovación Biosanitaria de Atención Primaria, Madrid, Spain; 2 Instituto de Investigación Sanitaria del Hospital Universitario la Paz (IdIPAZ), Madrid, Spain; 3 Red de Investigación en Servicios de Salud en Enfermedades Crónicas (REDISSEC), Madrid, Spain; 4 Subdirección General de Investigación Sanitaria y Documentación, Consejería de Sanidad, Madrid, Spain; 5 Centro de Salud Las Calesas, Gerencia Asistencial de Atención Primaria, Calle de Las Calesas, 12, 28026 Madrid, Spain; 6 Sistemas de Información, Gerencia Asistencial de Atención Primaria Madrid, Madrid, Spain; 7 Universidad Alfonso x El Sabio, Madrid, Spain; 8 Departamento de Medicina Interna, Hospital Universitario Clínico San Carlos, Facultad de Medicina, Universidad Complutense de Madrid (UCM), Instituto de Investigación Sanitaria Hospital Clínico de San Carlos (IdISSC), Madrid, Spain; 9 Hospital Central de la Defensa, Madrid, Spain; 10 Computational Biology Group, Precision Nutrition and Cancer Program, IMDEA Food Institute, Madrid, Spain; 11 Unidad Docente y de Investigación en Medicina Preventiva y Salud PúblicaFacultad de Ciencias de La Salud, Universidad Rey Juan Carlos, Alcorcón, Madrid Spain; 12 Departamento de Medicina Preventiva del Hospital Clínico de San Carlos, Instituto de Investigación Sanitaria Hospital Clínico de San Carlos (IdISSC), Madrid, Spain; 13 Departamento de Medicina Interna, Hospital General Universitario Gregorio Marañón. Facultad de Medicina, Universidad Complutense de Madrid (UCM), Instituto de Investigación Sanitaria Gregorio Marañón (IiSGM), Madrid, Spain

Correction to: *Scientific Reports* https://doi.org/10.1038/s41598-021-94121-8, published online 27 July 2021

The original version of this Article omitted the Funding section. The Funding section now reads:

“This study has been funded by Instituto de Salud Carlos III through the projects “PI15/00259” and "PI18/01025", and co-funded by a European Regional Development Fund, “A way of shaping Europe”

The original Article has been corrected.

