# Supplementary material for: Cardiovascular risk factors associated with acute myocardial infarction and stroke in the MADIABETES cohort
Source: Sci Rep. 2021 Jul 27;11:15245. doi: 10.1038/s41598-021-94121-8 (PMC8316319; doi:10.1038/s41598-021-94121-8)
Supplement: Supplementary file 1 — Supplementary Information. [file 41598_2021_94121_MOESM1_ESM.pdf]

Cardiovascular risk factors associated with acute myocardial infarction and stroke in the MADIABETES cohort

Salinero-Fort MA[1,2,3,4], San Andrés-Rebollo FJ[1,5,*], Cárdenas-Valladolid J[1,6,7], Méndez-Bailón M[8], Chico-Moraleja RM[9], Carrillo de Santa Pau E[10], Jiménez-Trujillo I[11], Gómez-Campelo P[2], de Burgos Lunar C[12], de Miguel-Yanes JM[13], MADIABETES**.

[1]Fundación de Investigación e Innovación Biosanitaria de Atención Primaria, Madrid, Spain.
[2]Instituto de Investigación Sanitaria del Hospital Universitario La Paz (IdIPAZ), Madrid, Spain.
[3]Red de Investigación en Servicios de Salud en Enfermedades Crónicas (REDISSEC), Madrid, Spain.
[4]Subdirección General de Investigación Sanitaria y Documentación. Consejería de Sanidad, Madrid, Spain.
[5]Centro de Salud Las Calesas. Gerencia Asistencial de Atención Primaria, Madrid, Spain.
[6]Sistemas de Información. Gerencia Asistencial de Atención Primaria Madrid, Spain.
[7]Universidad Alfonso X El Sabio, Madrid, Spain.
[8]Departamento de Medicina Interna, Hospital Universitario Clínico San Carlos. Facultad de Medicina, Universidad Complutense de Madrid (UCM). Instituto de Investigación Sanitaria Hospital Clínico de San Carlos (IdISSC), Madrid, Spain.
[9]Hospital Central de la Defensa, Madrid, Spain.
[10]Computational Biology Group. Precision Nutrition and Cancer Program. IMDEA Food Institute, Madrid, Spain.
[11]Unidad Docente y de Investigación en Medicina Preventiva y Salud Pública, Facultad de Ciencias de la Salud. Universidad Rey Juan Carlos, Alcorcón, Madrid, Spain.
[12]Departamento de Medicina Preventiva del Hospital Clínico de San Carlos. Instituto de Investigación Sanitaria Hospital Clínico de San Carlos (IdISSC), Madrid, Spain.
[13]Departamento de Medicina Interna, Hospital General Universitario Gregorio Marañón. Facultad de Medicina, Universidad Complutense de Madrid (UCM). Instituto de Investigación Sanitaria Gregorio Marañón (IiSGM), Madrid, Spain.
** A list of authors and their affiliations appears at the end of the paper.

Corresponding author:

San Andrés-Rebollo FJ
Centro de Salud Las Calesas
Gerencia Asistencial de Atención Primaria
Madrid. Spain.
E-mail: fcojavier.san@salud.madrid.org

Supplementary Table S1. Age-specific and total crude cumulative incidence of myocardial infarction stratified by sex

**Men**	Patients who had MI as the first cardiovascular event				**Women**	Patients who had MI as the first cardiovascular event			
Age group	No	Yes	Total	Crude Cumulative Incidence (x1000)	Age group	No	Yes	Total	Crude Cumulative Incidence (x1000)
<55	263	12	275	43.64	<55	165	5	170	29.41
55-59	170	6	176	34.09	55-59	133	2	135	14.81
60-64	214	11	225	48.89	60-64	192	4	196	20.41
65-69	205	11	216	50.93	65-69	233	8	241	33.20
70-74	218	6	224	26.79	70-74	295	8	303	26.40
75-79	165	13	178	73.03	75-79	284	12	296	40.54
80-84	89	2	91	21.98	80-84	150	7	157	44.59
>84	22	1	23	43.48	>84	68	6	74	81.08
Total	1346	62	1408	44.03	Total	1520	52	1572	33.08

Supplementary Table S2. Age-standardized incidence of myocardial infarction: total and stratified by age group and sex

**MEN**			**WOMEN**		
**Age Group**	**N**	**Incidence x 1000**	**Age Group**	**N**	**Incidence x 1000**
<55	445	43.64	<55	445	29.41
55-59	311	34.09	55-59	311	14.81
60-64	421	48.89	60-64	421	20.41
65-69	457	50.93	65-69	457	33.20
70-74	527	26.79	70-74	527	26.40
75-79	474	73.03	75-79	474	40.54
80-84	248	21.98	80-84	248	44.59
>84	97	43.48	>84	97	81.08

Age-standardized incidence in men: $(\Sigma (N_i * I_i) / \Sigma N_i) * 1000 = 44.39$

Age-standardized incidence in women: $(\Sigma (N_i * I_i) / \Sigma N_i) * 1000 = 31.38$

Supplementary Table S3. Age-specific and total crude cumulative incidence of stroke/TIA stratified by sex

**Men**	Patients who had a stroke as the first cardiovascular event				**Women**	Patients who had a stroke as the first cardiovascular event			
Age group	No	Yes	Total	Crude Cumulative Incidence (x1000)	Age group	No	Yes	Total	Crude Cumulative Incidence (x1000)
<55	272	3	275	10.91	<55	164	6	170	35.29
55-59	173	3	176	17.05	55-59	127	8	135	59.26
60-64	211	14	225	62.22	60-64	189	7	196	35.71
65-69	207	9	216	41.67	65-69	230	11	241	45.64
70-74	208	16	224	71.43	70-74	280	23	303	75.91
75-79	167	11	178	61.80	75-79	268	28	296	94.59
80-84	78	13	91	142.86	80-84	136	21	157	133.76
>84	20	3	23	130.43	>84	65	9	74	121.62
Total	1336	72	1408	51.14	Total	1459	113	1572	71.88

Supplementary Table S4. Age-standardized incidence of stroke/TIA: total and stratified by age group and sex

**MEN**			**WOMEN**		
**Age Group**	**N**	**Incidence x 1000**	**Age Group**	**N**	**Incidence x 1000**
<55	445	10.91	<55	445	35.29
55-59	311	17.05	55-59	311	59.26
60-64	421	62.22	60-64	421	35.71
65-69	457	41.67	65-69	457	45.64
70-74	527	71.43	70-74	527	75.91
75-79	474	61.80	75-79	474	94.59
80-84	248	142.86	80-84	248	133.76
>84	97	130.43	>84	97	121.62

Age-standardized incidence in men: $(\Sigma (N_i*I_i)/ \Sigma N_i)*1000$= 57.18

Age-standardized incidence in women: $(\Sigma (N_i*I_i)/ \Sigma N_i)*1000$= 67.06

Supplementary Fig. S1. Modeling process

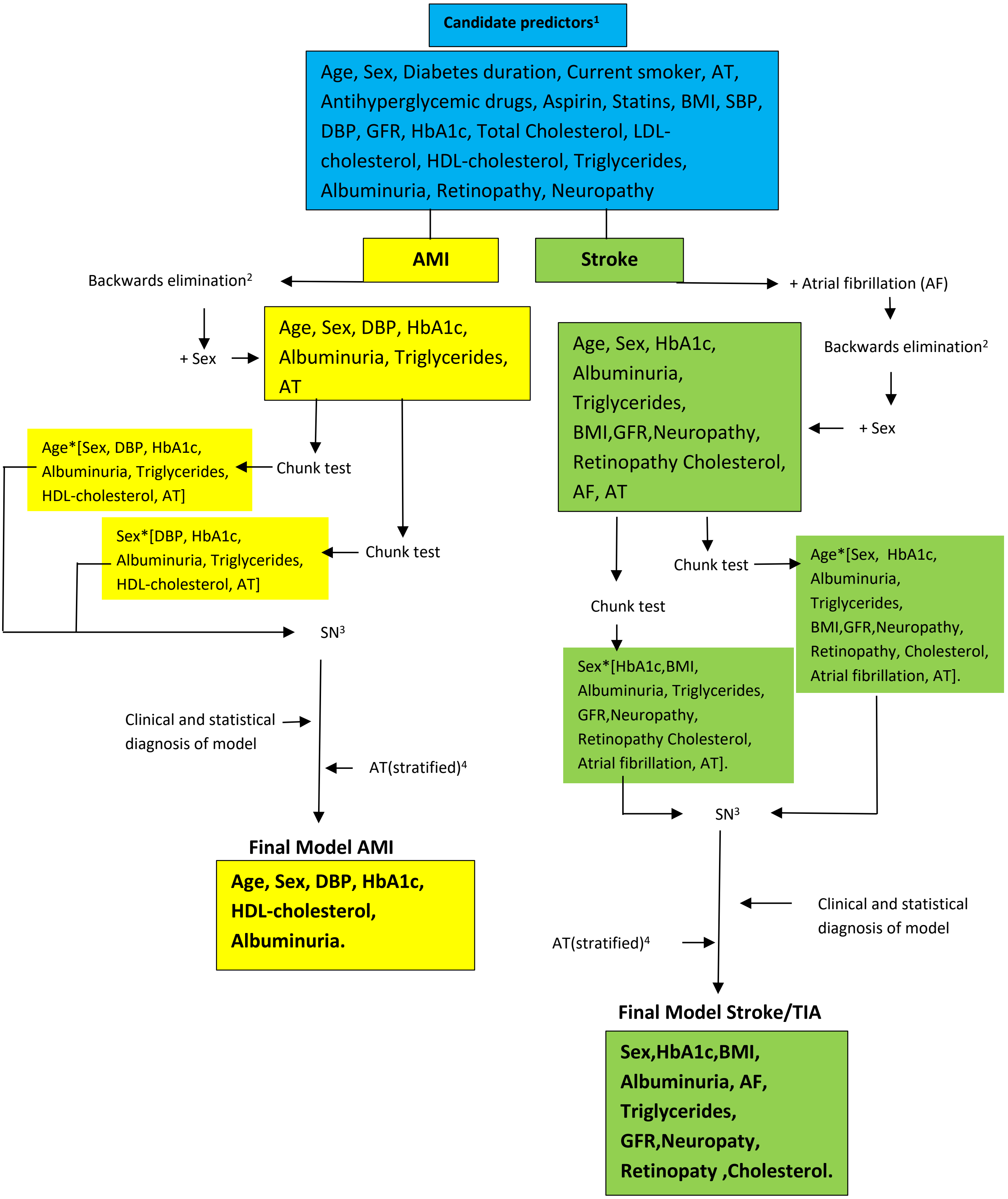

1-AT: Antihypertensive treatment. BMI: Body mass index. SBP: Systolic blood pressure. DBP: Diastolic blood pressure. GFR: Glomerular filtration rate. HbA1c: Glycated haemoglobin, HDL-cholesterol: High-density lipoprotein cholesterol, LDL-cholesterol: Low-density lipoprotein cholesterol, AMI: Acute myocardial infarction. TIA: Transient ischemic attack. AF: atrial fibrillation.

2- Probability for variable entry (PIN=0.05). Probability of likelihood ratio to remove a variable (POUT=0.2)

3- Statistically non-significant.

4- The inclusion as an independent variable produced inconsistent results given that the vast majority of the cardiovascular events were concentrated in the category of patients taking antihypertensive drugs. We repeated the analysis stratifying by this variable that allows taking it into account in the adjustment and solving this problem.

Supplementary Fig. S2. Continuous predictors that did not meet the assumption of log-linear relationship. Acute myocardial infarction model*

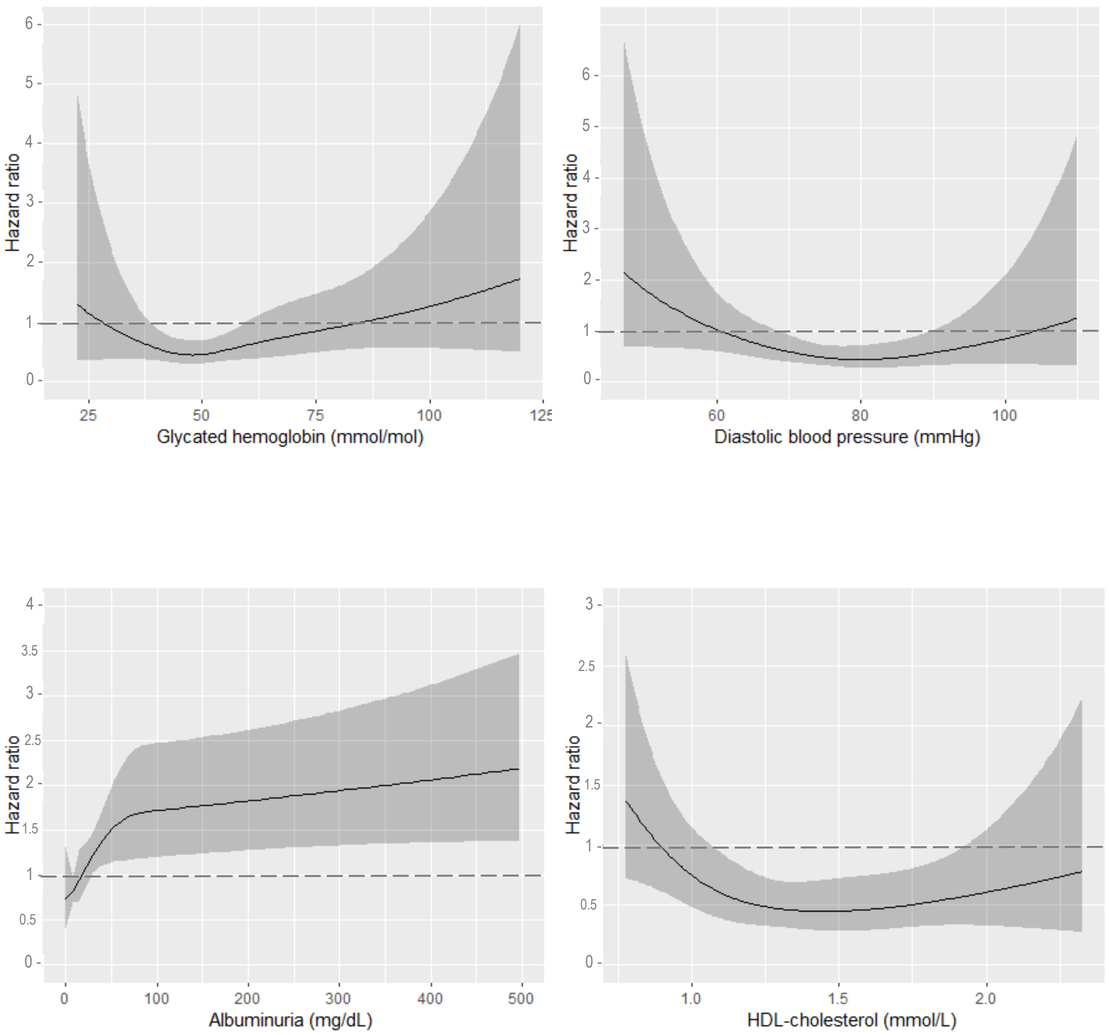

*Curves are shown for restricted cubic spline functions with 4 knots Each variable is adjusted for all variables (mean value) included in the final model.

Supplementary Fig. S3. Continuous predictors that did not meet the assumption of log-linear relationship. Stroke/transient ischemic attack model*

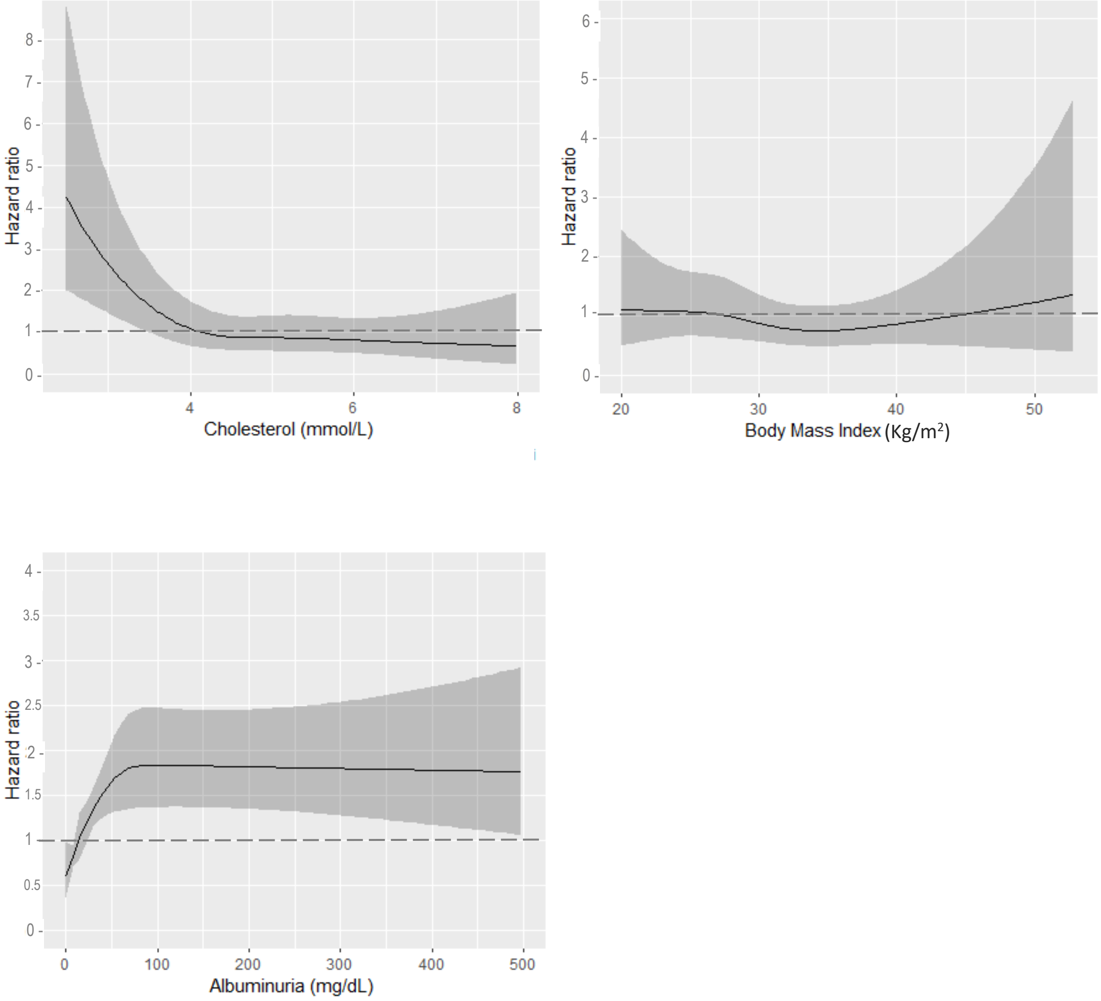

*Curves are shown for restricted cubic spline functions with 4 knots. Each variable is adjusted for all variables (mean value) included in the final model.
